# Predictors of transient congenital primary hypothyroidism: data from the German registry for congenital hypothyroidism (AQUAPE “HypoDok”)

**DOI:** 10.1007/s00431-021-04031-0

**Published:** 2021-03-25

**Authors:** Nicola Matejek, Sascha R. Tittel, Holger Haberland, Tilman Rohrer, Eva-Maria Busemann, Norbert Jorch, Karl-Otfried Schwab, Joachim Wölfle, Reinhard W. Holl, Markus Bettendorf

**Affiliations:** 1 Division of Paediatric Endocrinology and Diabetes, Department of Paediatrics, University Children’s Hospital Heidelberg, Im Neuenheimer Feld 430, 69120 Heidelberg, Germany; 2 Central Institute for Biomedical Technology, Institute for Epidemiology and Medical Biometry, University of Ulm, Ulm, Germany; 3 Social-Paediatric Centre, Sana-Hospital Lichtenberg, Berlin, Germany; 4 Department of Paediatrics, University Hospital Homburg-Saar, Homburg, Germany; 5 Catholic Children’s Hospital Wilhelmstift, Hamburg, Germany; 6 Department of Paediatrics, Evangelical Hospital Bethel, Bielefeld, Germany; 7 Division of Paediatric Endocrinology and Diabetes, University Hospital Freiburg, Freiburg, Germany; 8 Department of Paediatrics, University Hospital Erlangen, Erlangen, Germany

**Keywords:** Congenital primary hypothyroidism, Prediction, Transient congenital primary hypothyroidism, Permanent congenital primary hypothyroidism

## Abstract

Neonatal screening for congenital primary hypothyroidism (CH) may not distinguish between transient (TCH) and permanent dysfunction (PCH), causing potential overtreatment and concerns in affected families. To specify the indication for interruption of therapy, we analysed the German registry “HypoDok” for infants with CH, which oversees 1625 patients from 49 participating centres in Germany and Austria from 1997 until today. A total of 357 patients with a thyroid gland in loco typico were identified and retrospectively grouped according to cessation (TCH, *n* = 24) or continuation (PCH, *n* = 333) of l-thyroxine (l-T_4_) treatment at 2 years of age. The receiver operating characteristic (ROC) analysis was performed to identify cutoffs predicting TCH by screening TSH concentrations and l-T_4_ dosages. Gestational ages, birth weights and prevalence of associated malformations were comparable in both groups. The cutoff screening TSH concentration was 73 mU/L. The cutoff daily l-T_4_ dosage at 1 year was 3.1 μg/kg (90% sensitivity, 63% specificity; 36 μg/day) and at 2 years of age 2.95 μg/kg (91% sensitivity, 59% specificity; 40 μg/day). At 2 years of age, specificity (71%) increased when both of these parameters were considered together.

*Conclusion*: The decision to continue or cease l-T_4_ treatment at 2 years of age in CH patients diagnosed in neonatal screening may be based on their screening TSH concentrations and individual l-T_4_ dosages at 1 and 2 years of age. Thus, TCH and PCH may be distinguished; overtreatment avoided; and affected families reassured.

**Table:** 

**What is Known:** • *The course of congenital primary hypothyroidism may be transient, causing potential overtreatment.* • *The dose of* *l-thyroxine at 1 or 2 years of age may predict a transient course of primary congenital hypothyroidism.*	
**What is New:** • *TSH screening concentration and* *l-thyroxine dosages at 1 and 2 years of age represent reliable predictors for transient congenital primary hypothyroidism with higher sensitivity and specificity when considered together in order to select eligible patients who qualify for treatment withdrawal.*

**What is Known:**

• *The course of congenital primary hypothyroidism may be transient, causing potential overtreatment.*

• *The dose of* *l-thyroxine at 1 or 2 years of age may predict a transient course of primary congenital hypothyroidism.*

**What is New:**

• *TSH screening concentration and* *l-thyroxine dosages at 1 and 2 years of age represent reliable predictors for transient congenital primary hypothyroidism with higher sensitivity and specificity when considered together in order to select eligible patients who qualify for treatment withdrawal.*

## Introduction

Congenital primary hypothyroidism (CH) is suspected in neonatal screening when capillary TSH concentrations are elevated (> 15 mU/L in Germany). The diagnosis is confirmed by measuring venous TSH and fT_4_ concentrations before the start of treatment [1]. Not all infants with confirmed CH necessarily receive lifelong l-T_4_ treatment. Transient congenital hypothyroidism (TCH) occurs in up to 35% of children with CH [2]. Lowering the threshold screening TSH concentrations for diagnosing CH may suggest an increased prevalence, overtreatment and impaired outcome in children that only have transient or mild hypothyroidism [3]. Gene mutations of *DUOX2* and *TSH-R* have been described in cases with mild transient hypothyroidism [4–7]. National and international guidelines recommend confirming CH after the second birthday in case an unequivocal diagnosis has not been established during the neonatal period. l-T_4_ treatment is then paused for 4 to 6 weeks in order to assess endogenous thyroid function. Earlier withdrawal is discussed when transient elevations of neonatal TSH concentrations are likely and there is impending overtreatment [7–10]. Paediatric endocrinologists tend to conduct therapy in the first 2 years of life in order to avoid defects in the myelinisation of the central nervous system and to assure normal neurodevelopmental outcomes. A re-evaluation of thyroid function is indicated if the thyroid gland is developed normally and elevated TSH serum levels are not observed or there has been no need to adjust the dosage of l-T_4_ during the course of treatment.

However, standard recommendations for interruption of treatment are lacking [11].

We analysed data from the German registry of CH in order to determine whether screening and serum TSH concentrations and l-T_4_ dosages at 1 and 2 years of age are sufficient parameters to anticipate a transient nature of thyroid dysfunction warranting its re-evaluation.

## Methods

“HypoDok” is a prospective documentation software for CH supported by the German Society of Paediatric Endocrinology and Diabetes (DGKED), with contributions from 49 participating centres in Germany and Austria currently including 1625 patients. The inclusion criteria were the availability of screening TSH concentrations (mU/L), a thyroid gland in loco typico, visualised by ultrasound, and the l-T_4_ dosages (μg/kg/day, μg/day) at diagnosis and at 1 and/or 2 years of age, respectively. The end of l-T_4_ treatment was documented by checking a corresponding box on the date of withdrawal. The following items were extracted from the registry: l-T_4_ dosages at 6 months of age, weeks of gestation, birth weight (g), Apgar-Score, age at measurement of screening TSH and of serum TSH, serum TSH (mU/L) and fT_4_ (ng/dL) concentrations at confirmation, as well as relevant maternal and patient’s history (selection options: yes/no): gender male, maternal hypothyroidism, maternal treatment with l-T_4_ during pregnancy, hyperthyroidism, anti-thyroid drugs (ATD) during pregnancy, iodine medication in pregnancy and delivery, diagnosis of Trisomy 21 and dopamine treatment of the neonate. Additional diagnoses or malformations captured as free text documentation were also considered in the analyses. The height (cm) and body mass index (kg/m^2^) expressed as standard deviation scores (SDS) [12] at the age of 2 years, the l-T_4_ withdrawal period of 4 to 6 weeks and the results of psychomotor testing at the age of 2 years were extracted. l-T_4_ dosage changes were collected from each visit. The screening TSH concentrations were measured in dry-blood spots by the regional neonatal screening laboratories in mU/L. The serum TSH (mU/L) and fT_4_ (ng/dL) concentrations were measured in the laboratory of the respective centre for paediatric endocrinology. A total of 357 patients treated in 37 German centres were eligible and were grouped according to continuation of l-T_4_ beyond the 2nd year of life (PCH) or cessation (TCH) of l-T_4_ treatment within the first 2 years of life.

### Statistics

Descriptive data were presented as the median and interquartile range for continuous values and percentage for binomial/categorical values. Wilcoxon’s rank sum test was used to compare continuous variables between groups, while nominal variables were analysed by chi-squared test. The results were considered significant at *p* < 0.05. The receiver operating characteristic (ROC) analysis was performed to identify cutoffs predicting TCH by screening TSH concentrations and l-T_4_ dosages (μg/kg/day and μg/day) at 6 month and 1 and 2 years of age, respectively. We used SAS 9.4 (SAS Inc., Cary, NC, USA) and PROC LOGISTIC to calculate predicted probabilities of the patients to belong either to the TCH or PCH group, as well as their sensitivity and specificity based on the respective screening TSH concentration or l-T_4_ dosage. The optimal cutoff for each parameter was calculated by maximising the Youden index [13]. Using linear regression made differences of screening TSH between patients with and without l-T_4_ withdrawal period, means are presented as least square means with 95% confidence interval.

## Results

**Fig. 1:**
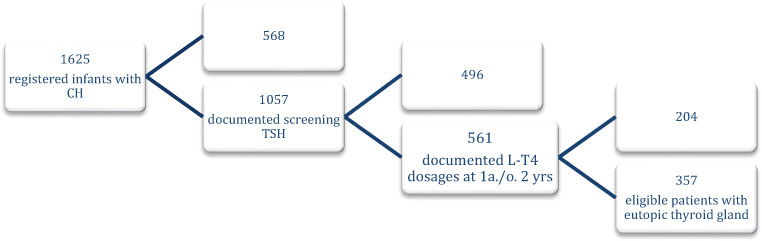
Selection of eligible patients according to the inclusion criteria: screening TSH concentrations, a eutopic thyroid gland visualised by ultrasound and the l-T_4_ dosages at diagnosis and at 1 and 2 years of age

A total of 357 infants with congenital primary hypothyroidism met the inclusion criteria (Fig. 1). They were grouped retrospectively as PCH (*n* = 333) and TCH (*n* = 24) (Table 1). All patients with TCH terminated therapy after 2 years of age (24/24). 95.2% of patients with PCH temporarily paused l-T_4_ treatment for 4 to 6 weeks (*n* = 111, 33%) and had to continue the treatment afterwards and/or required an increase of l-T_4_ dosage during the treatment course (*n* = 316, 95%). Screening TSH concentrations tended to be lower in neonates with TCH (55.8 mU/L) than in those with PCH (150.0 mU/L, *p* = 0.06), whereas serum TSH and fT_4_ concentrations were similar at confirmation of the diagnosis (Table 2). Neonatal screening was done at 3 days of age in both groups and the confirmation of the diagnosis at 11 (PCH) and 13.5 days of age (TCH; Table 2), respectively. l-T_4_ dosages at start of therapy in PCH and in TCH were comparable (*p* = 1.0). l-T_4_ dosages per kilogramme body weight at 6 months of age were similar, and receiver operating characteristic calculation revealed 27 μg/day as predicting cutoff for TCH (sensitivity 77%, specificity 54%). At 1 year of age, the l-T_4_ dosages were significantly higher in PCH (4.52 μg/kg/day, total dose 45 μg/day) than in TCH (2.96 μg/kg/day, *p* < 0.01; 30 μg/day, *p* < 0.01), and were also higher at 2 years of age in PCH (4.03 μg/kg/day, 50 μg/day) than in TCH (2.5 μg/kg/day, *p* < 0.01; 37 μg/day, *p* < 0.01) (Table 2).

**Table 1:** Demographic characteristics and medical history of patients at diagnosis: patients were grouped as permanent CH (PCH; *n* = 333) or transient CH (TCH; *n* = 24). Variables are presented as median and interquartile range (A) or prevalence (%; B)

	PCH			TCH				
A								
Variable	*n*	Median	Interquartile range	*n*	Median	Interquartile range		*p*
Weeks of gestation	333	40	38–41	24	40	37–40		1.0
Birth weight (g)	325	3420	3060–3800	22	3380	2920–3850		1.0
APGAR-score	326	10	7–10	22	10	9–10		1.0
BMI (kg/m^2^) at 2 years	301	16.6	15.6–17.5	22	16	14.7–16.5		0.8
BMI-SDS at 2 years	301	0.4	0.3–1.0	22	0.0	− 1.1–0.4		0.7
Height-SDS at 2 years	301	0.1	− 0.6–0.8	23	0.0	− 0.6–0.5		1.0
B								
Variable	*n*		Prevalence (%)		*n*	Prevalence (%)		*p*
Weeks of gestation < 36	322		5.6	-	24	12.5	-	1.0
Male gender	333		35.1	-	24	50	-	1.0
Trisomy 21	333		1.2	-	24	4.2	-	1.0
Dopamine therapy	333		0.9	-	24	8.3	-	0.07
ATD in pregnancy	101		2	-	11	18.2	-	0.1
l-T_4_ withdrawal period	211		52.6	-	24	100	-	<0.01
l-T_4_ withdrawal period a./o. l-T_4_ dosage increase	333		95.2	-	24	100	-	1.0
Developmental test: normal	141		89	-	8	100	-	1.0

**Table 2:** Screening TSH (mU/L), confirmation serum TSH (mU/L), serum fT_4_ (ng/mL), age at neonatal screening and confirmation (days) and dosages of l-T_4_ at diagnosis, at the age of 6 months and 1 and 2 years in patients with permanent CH (PCH, *n* = 273) and transient CH (TCH, *n* = 23). Data are given as median with interquartile range; *p* < 0.05 was considered significantly different by *χ*^2^ test

	PCH			TCH			
Variable	*n*	Median	Interquartile range	*n*	Median	Interquartile range	*p*
Screening TSH (mU/L)	333	150	62–237	24	55.8	32.5–136	0.06
Age at neonatal screening	305	3	2–5	24	3	2–8.5	1.0
Age at confirmation diagnosis	333	11	6–20	24	13.5	10.5–18	1.0
Confirmation TSH (mU/L)	257	118	62–227	23	100	42–169	1.0
Serum fT_4_ (ng/dL)	275	1.16	0.5–2.33	21	0.96	0.4–1.4	1.0
l-T_4_ dosage at diagnosis (μg)	333	50	44–50	24	50	29–50	1.0
l-T_4_ dosage at diagnosis (μg/kg)	328	13.2	10–15	23	12.5	10–14	1.0
l-T_4_ dosage at 6 months (μg)	157	38	30–50	13	25	25–38	0.4
l-T_4_ dosage at 6 months (μg/kg)	153	4.74	3.8–5.76	13	4.47	3.25–5	1.0
l-T_4_ dosage at 1 year (μg)	307	45	38–50	24	30	25–42	<0.01
l-T_4_ dosage at 1 year (μg/kg)	306	4.52	3.8–5.2	24	2.96	2.3–4.6	<0.01
l-T_4_ dosage at 2 years (μg)	302	50	44–60	23	37	25–50	<0.01
l-T_4_ dosage at 2 years (μg/kg)	301	4.03	3.6–4.74	22	2.5	1.95–3.66	<0.01

Infants with a l-T_4_ withdrawal period had significant lower screening TSH: 142.6 mU/L (119.2–166) vs. 186.2 mU/L (159–213.4, *p* = 0.02), shown by linear regression analysis (Table 3).

**Table 3:** Subgroup analyses in patients with screening TSH < 73mU/L (*n* = 109): l-T_4_ dosages at the age of 6 months and 1 and 2 years in patients with permanent CH (PCH, *n* = 94) and transient CH (TCH, *n* = 15)

	PCH			TCH			
Variable	*n*	Median	Interquartile range	*n*	Median	Interquartile range	*p*
l-T_4_ dose at 6 months (μg)	45	35	25–38	10	25	25–35	0.35
l-T_4_ dose at 6 months (μg/kg)	45	4.1	3.4–5.4	10	3.6	3.2–4.5	0.5
l-T_4_ dose at 1 year (μg)	86	40	37–50	15	25	25–44	0.02
l-T_4_ dose at 1 year (μg/kg)	86	4.4	3.5–5.2	15	2.8	2.3–4.9	0.04
l-T_4_ dose at 2 years (μg)	82	50	38–50	14	25	25–40	<0.01
l-T_4_ dose at 2 years (μg/kg)	82	3.9	3.2–4.3	14	2.3	1.95–3.7	0.01
l-T_4_ withdrawal period a./o. l-T_4_ dosage increase	94	94	-	15	100	-	0.5

**Fig. 2:**
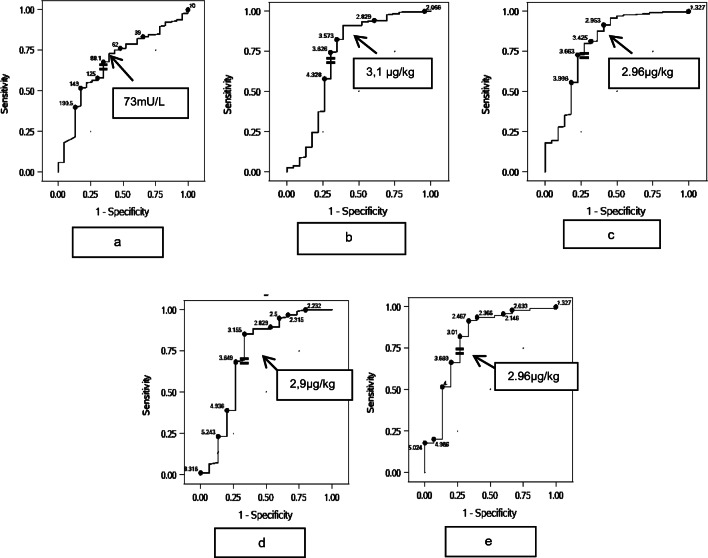
Receiver operating characteristic (ROC) for screening TSH concentration (**a**), dosages of l-T_4_ at the age of 1 (**b**) and 2 (**c**) years in patients with CH predicting TCH. Subgroup analyses in patients with screening TSH < 73 mU/L: dosages of l-T_4_ at the ages of 1 (**d**) and 2 (**e**) years

The cutoff screening TSH concentration by ROC was 73 mU/L (Fig. 2a). The cutoff l-T_4_ dosage at 1 year of age was 3.1 μg/kg/day (Fig. 2b) and 2.95 μg/kg/day after 2 years (Fig. 2c) (Table 4, A). The l-T_4_ dosage with 99% sensitivity was 2.0μg/kg/day (20 μg/day) and 6.3 μg/kg/day (60 μg/day) with 96% specificity at 1 year of age. At 2 years of age, the l-T_4_ dosage of 2.0 μg/kg/day (25 μg/day) was 99% sensitive for TCH and 5.0 μg/kg/day (55 μg/day) was 96% specific for PCH (Table 4, A).

**Table 4:** Cutoff concentrations predicting TCH by screening TSH concentration and dosages of l-T_4_ at the ages of 1 and 2 years (A). Subgroup analyses in patients with screening TSH < 73 mU/L (PCH *n* = 94, TCH *n* = 15) (B)

	Cutoff	Sensitivity %	Specificity %
A			
Screening TSH	73 mU/L	72	63
l-T_4_ at 1 year μg/kg/day	**3.1**	**90**	**63**
μg/day	**36**	**85**	**54**
l-T_4_ at 1 year μg/kg/day	2.0	99	4
μg/day	20	99	8
l-T_4_ at 1 year μg/kg/day	6.3	4	96
μg/day	60	10	96
l-T_4_ at 2 years μg/kg/day	**2.95**	**91**	**59**
μg/day	**40**	**84**	**65**
l-T_4_ at 2 years μg/kg/day	2.0	99	27
μg/day	25	100	13
l-T_4_ at 2 years μg/kg/day	5.0	18	96
μg/day	55	31	96
B			
l-T_4_ at 1 year μg/kg/day	**2.9**	**85**	**60**
μg/day	**27.5**	**86**	**60**
l-T_4_ at 1 year μg/kg/day	2.2	99	13
μg/day	20	99	7
l-T_4_ at 1 year μg/kg/day	6.6	2	93
μg/day	60	8	100
l-T_4_ at 2 years μg/kg/day	**2.96**	**81**	**71**
μg/day	**38**	**83**	**64**
l-T_4_ at 2 years μg/kg/day	1.85	99	21
μg/day	25	99	7
l-T_4_ at 2 years μg/kg/day	6.3	11	100
μg/day	72.5	18	100

In a subgroup with screening TSH concentration below 73 mU/L (*n* = 109), the proportion of TCH (16%) was twice as high as in the total cohort (PCH, *n* = 94 vs. TCH, *n* = 15). The total l-T_4_ dosage at the age of 1 year (27.5 μg/day, 2.9 μg/kg/day) (Table 4, B; Fig. 2d) and at the age of 2 years (38 μg/day, 2.96 μg/kg/day) (Table 4, B; Fig. 2e) predicted a transient CH course with a slightly lower sensitivity at 1 year and similar sensitivity at 2 years with more specificity (71%). Predicting l-T_4_ dosages with highest sensitivity and specificity were slightly higher for TCH (2.2 μg/kg/day) and for PCH (6.6 μg/kg/day) at 1 year of age in this subgroup compared to the overall group. The l-T_4_ dosage with the highest sensitivity for TCH at 2 years of age is lower than in the entire group (1.85 μg/kg/day), but the total daily l-T_4_ dosage is identical, as well as the dosage for the highest specificity. Similar to the overall group, we suggest a TCH predicting l-T_4_ dosage of 27.5 μg/kg/day (sensitivity 62% and specificity 70%) at 6 months of age.

The demographic characteristics of patients are listed in Table 1, B. Maternal hypothyroidism and treatment with l-T_4_ during pregnancy were similar in both groups as well as the number of infants with other congenital malformations (data not shown). Exposure to iodine medication during pregnancy or delivery was comparable in both groups (data not shown). Neonates with TCH were more frequently treated with dopamine than those with PCH (8.3% vs. 0.9%, *p* = 0.07) and mothers of neonates with TCH were more often treated with ATD (*p* = 0.1). An increase of the l-T_4_ dosage was required in almost all of PCH patients (Table 1, B) while l-T_4_ withdrawal was only carried out in one-third of PCH patients.

At 2 years of age, median heights and BMI of all patients with TCH and PCH were similar (*p* = 1.0, *p* = 0.8) (Table 1, A). The results of developmental tests were documented in 141/333 PCH and in 8/24 TCH patients and revealed normal results in 89% and 100% (*p* = 1.0) of patients, respectively.

## Discussion

In this study, we assessed screening and serum TSH concentrations and dosages of l-T_4_ at 6 months and 1 and 2 years of age in infants with CH and a eutopic thyroid gland registered in “HypoDok” in order to predict transient or permanent hypothyroidism. A total of 160 to 280 patients with CH are detected in the neonatal screening in Germany per year [14], of which about 18% are registered in “HypoDok”. In Germany, it is not mandatory to register patients for treatment.

Detection of milder forms of CH has refocused attention on the initial intent of neonatal screening, namely prevention of mental retardation. Lowering the threshold of TSH concentrations in the neonatal screening prompted an increase of positive CH results [15] and more cases with mild hypothyroidism and transient courses were detected [15, 16]. The decrease of the TSH threshold in all likelihood increased the laboratory and economic burden of neonatal screening programs as well as the concern of affected families, but it is not clear whether these patients actually benefit from early detection and treatment [17–19]. A lower TSH threshold in the neonate screening in other countries outside of Germany (> 15 mU/L) could explain the higher percentage of TCH in other studies [19, 20]. Retrospective studies showed that neonates with a mildly elevated screening TSH (< 15 and < 20 mU/L) are at risk for permanent hypothyroidism [3, 9, 19]. As up to 35% of patients may be affected by TCH, defining these criteria seems worthwhile. Our analyses revealed a rate of 7% for TCH, which is lower than reported in previous studies [2, 8, 9, 16]. In order not to treat infants with TCH unnecessarily for too long, a safe approach for infants with TCH should be defined in guidelines [1, 20, 21]. Current guidelines recommend re-evaluation of the thyroid axis after 2 years of age and after completion of CNS myelination [1, 20] but concise evaluation criteria for this are lacking so far [22, 23]. The current recommendations of the 2020 consensus congenital hypothyroidism guideline update may raise the prevalence of TCH, as treatment of hyperthyreotropinemia is recommended from the second week of life [21]. The differentiation of isolated hyperthyreotropinemia and primary hypothyroidism in neonates proves challenging [23].

Reliable predictors represent the basis for the recommendation to withdraw l-T_4_ in infancy when the diagnosis of hypothyroidism remains uncertain for neonates with a normally located thyroid gland.

Serum TSH concentrations at diagnosis were similar for all patients with TCH and PCH, which confirms previous analyses on discrimination between TCH and PCH in children with a eutopic thyroid gland [24, 25]. Neonatal screening is scheduled for a narrow period of time within 14 days of age [1, 21] and is conducted in 11 German screening laboratories. Therefore, age-dependent variations of serum TSH concentrations are expected to have a minor effect as in the vast majority of cases the confirmation of diagnosis was done within the first 14 days of age in both groups. Serum TSH concentrations may be further affected by daytime, gender [26] and specific assay modalities such as range and sensitivity [27].

Treatment of mothers with iodine, anti-thyroid drugs or dopamine medication in neonates frequently causes TCH, because these drugs suppress thyroid function in the neonate temporarily [26, 27]. In our study, in the TCH group, dopamine medication was more often used in the neonatal period as expected. The prevalence of TCH and PCH was similar in our analyses when mothers were treated with anti-thyroid drugs during pregnancy, but insufficiently treated Morbus Basedow is a rare disease during pregnancy (prevalence 1:100.000–1:310.000 neonates) [28]. The proportion of preterm infants in our analysis is comparable to the overall premature birth rate in Germany [29]. Premature neonates have a higher risk of TCH, mediated by immaturity and medications during the intensive care period [19]. Thus, these cases will not to be reported to “HypoDok”, if temporary treatment is expected.

l-T_4_ treatment dosages at various time points during the first 3 years have been reported to discriminate TCH from PCH [2, 7–10, 16]. Based on these parameters, the decision to withdraw l-T_4_ treatment in infancy in order to re-evaluate endogenous thyroid function may be made.

When calculating the exact l-T_4_ dosage per kg body weight, the available galenic preparations and their strengths should be considered; the smallest incremental change in l-T_4_ dosage possible to prescribe is 5 μg/drop or 2 μg/0.1 mL liquid. Our results add to those of other groups who examined infants with CH and a eutopic thyroid gland [2, 7, 8, 16]. Our findings suggest the predicting cutoff for TCH at 2 years of age is the l-T_4_ dosage of 2.0 μg/kg/day and accordingly 25 μg/day, whereas results of other study groups ranged from 0.94 [8] to 2.8 μg/kg/day [16].

It is important to note that, based on our results, the screening TSH cutoff of 73 mU/L may be used as a discriminatory parameter, but this does not apply to the confirmatory TSH measurement. Confirmatory TSH concentration measured at around 11–14 days of age did not differ between the TCH and PCH group (Table 2).

The screening TSH may aid as a valid parameter for predicting TCH, as the sensitivity for screening TSH ≤ 73 mU/L shown by ROC is reliable and the impact of low screening TSH to decide on l-T_4_ withdrawal period is significant. Considering the l-T_4_ dosages at 1 and 2 years of age in infants with CH and a eutopic thyroid gland can make more reliable prediction of TCH and PCH. We are the first to report that a combination of both parameters increases the sensitivity and specificity of predicting either TCH or PCH.

This study has limitations due to the retrospective study design, the limited number of patients resulting from the limited overall CH patient coverage of “HypoDok” and the potential selection bias of patients included in the optional registry by the treating physicians. The optional participation in the “HypoDok” registry results in incomplete documentation and reduces the number of eligible patients. A register analysis is not allowed to publish cutoff values with the highest sensitivity predicting TCH and with the highest specificity predicting PCH, because the anonymity of patient data may be compromised. Therefore, we present values with reliability over 95% or median values of 100%. However, a large population could be analysed, reflecting routine CH patient care in Germany. Thus, our results can provide a basis for the selection of those CH patients who qualify for treatment cessation in infancy. Overtreatment can influence the physical, neurological or behavioural development of young infants with lifelong consequences and may increase uncertainties for both families and physicians [15, 30]. Future studies aimed to confirm these parameters as prognostic markers for TCH should be planned prospectively and molecular analyses should be included.

## Data Availability

All data relevant to this study are included in the manuscript.

## Abbreviations

ATD: Anti-thyroid drugs
BMI: Body mass index
CH: Congenital primary hypothyroidism
DGKED: German Society of Paediatric Endocrinology and Diabetes
fT_4_: Free serum thyroxine
HypoDok: Specialised prospective documentation software for CH
l-T_4_: l-Thyroxine
PCH: Permanent congenital primary hypothyroidism
ROC: Receiver operating characteristic
SDS: Standard deviation score
TCH: Transient congenital primary hypothyroidism
TSH: Thyroid-stimulating hormone

## Code availability

SAS 9.4 (SAS Inc., Cary, NC, USA) and PROC LOGISTIC.
